# mSphere of Influence: a Community To Study Communities

**DOI:** 10.1128/mSphere.00047-20

**Published:** 2020-02-05

**Authors:** Dominique Limoli

**Affiliations:** a Microbiology and Immunology, University of Iowa, Iowa City, Iowa, USA

**Keywords:** *Pseudomonas aeruginosa*, cystic fibrosis, lung infection, microbial communities

## Abstract

Dominique Limoli studies polymicrobial interactions during cystic fibrosis respiratory disease. In this mSphere of Influence article, she reflects on how two papers (D. A. Hogan, S. D. Willger, E. L. Dolben, T. H. Hampton, et al., PLoS One 11:e0149998, 2016, https://doi.org/10.1371/journal.pone.0149998, and P. Jorth, B. J. Staudinger, X. Wu, K. B. Hisert, et al., Cell Host & Microbe 18:307–319, 2015, https://doi.org/10.1016/j.chom.2015.07.006) have influenced her thinking and research direction, which aims to understand interspecies bacterial communication during airway infections. These studies highlighted for her a need for new perspectives on the pathology of chronic infections in order to improve interventions.

## Abstract (precis)

Dominique Limoli studies polymicrobial interactions during cystic fibrosis respiratory disease. In this mSphere of Influence article, she reflects on how two papers (D. A. Hogan, S. D. Willger, E. L. Dolben, T. H. Hampton, et al., PLoS One 11:e0149998, 2016, https://doi.org/10.1371/journal.pone.0149998, and P. Jorth, B.

“I alone cannot change the world, but I can cast a stone across the waters to create many ripples.”

—Mother Teresa

## COMMENTARY

Why are chronic infections, such as those seen in the lungs of cystic fibrosis (CF) patients, so difficult to treat? When considering this question, the problem can often feel overwhelming, because, as with most things, it becomes clear that no one answer is satisfactory. Not only are chronic infections often composed of multiple microbial species, but significant phenotypic and genotypic heterogeneity are often found within species (1). Environmental factors additionally impose physical, nutritional, and chemical barriers and gradients which may further diversify communities, in both space and time (2). During CF pulmonary infection, this complexity often complicates efforts to address even the most basic and fundamental issues, such as which bacterial species are present in the airways and which organisms, or cohorts of organisms, are responsible for pulmonary damage and disease outcomes.

Through careful study of the respiratory secretions from individuals with CF, it is well appreciated that infections are most often composed of multiple microbial species (3). However, we lack consensus regarding how these species are spatially organized in the airways and thus the extent to which they interact in a biologically meaningful way. Regions of the lungs are known to be heterogeneous, a feature thought to be augmented in the CF airway due to extensive mucous plugging, resulting in differing oxygen and carbon concentrations and uneven distribution of inhaled antibiotic concentrations achieved in different regions of the airway. For example, it is commonly observed that patients experience earlier and more-severe lung damage in the upper right lobes (4). Does this damage result from the presence of a specific pathogen or collection of pathogens? Alternatively, does this altered lung environment select for colonization by a specific community?

As we consider the countless factors influencing the efficacy of efforts to treat or prevent chronic infections, many of which are challenging to reconstitute in the laboratory, it becomes clear that we require a greater understanding of the extrinsic and intrinsic factors shaping microbial communities and driving tolerance to treatments in their native environment. While we remain far from answering these questions, the extraordinary collective efforts made by the field to address them, in the face of such obstacles, inspire me to push the boundaries of my own research. Here, I am privileged to highlight two such studies which have had a particular influence on how I think about addressing these fundamental issues.

In the first study, entitled “Analysis of lung microbiota in bronchial alveolar lavage, protected brush and sputum samples from subjects with mild-to-moderate cystic fibrosis lung disease,” Deb Hogan and colleagues performed regional sampling of different lobes of the lungs of nine individuals with CF through bronchial alveolar lavage and protected brush sampling (5). The microbial communities were analyzed using DNA extracted from these respiratory samples and the extent of regional lung damage analyzed by computed tomography. Surprisingly, the extent of lung damage did not correlate with the presence or absence of specific genera, community diversity, composition, or abundance. Further, in all the subjects, BAL fluid from different regions of the lung had the same microbial community composition, arguing against the hypothesis that distant regions of the lung house divergent communities and that infecting species are spatially separate in the airway.

From these observations, the authors concluded that the absence of a significant link between regional lung damage and microbial community composition/abundance suggests that it may be the host-microbe interaction, rather than the nature of the microbial species present, that promotes lung damage. Alternatively, geographical differences may instead influence the phenotypic behavior of the organism present and not the composition of community members *per se*. This idea is further supported by studies tracking CF patients over the course of exacerbations, finding no clear or consistent pattern in community structure or overall bacterial load that could explain the onset of exacerbation (6–9).

In the second study, entitled “Regional isolation drives bacterial diversification within cystic fibrosis lungs,” Peter Jorth and colleagues sought further insight into these ideas by asking the following two fundamental questions (10). (i) Do clonally related isolates of Pseudomonas aeruginosa in different regions of chronically infected CF lungs differ functionally in ways that could affect their pathogenic potential? (ii) Does the isolation of bacteria in different regions of the lung contribute to a divergence of community genotypes or phenotypes?

To address these questions, the authors embarked on the enormous task of sterilely dissecting the upper, middle, and lower lobes of the lungs of eight patients with CF at the time of transplantation. Approximately 12,000 resident P. aeruginosa isolates were collected and analyzed for phenotypic, proteomic, and genomic differences. Their analyses revealed significant regional differences in the genotypes and phenotypes present. For example, the right upper lobe of patient 1 contained mostly ciprofloxacin-resistant isolates, whereas the isolates from the right lower lobe were sensitive. Proteomic analysis also revealed that isolates from different regions were functionally more similar to each other than to those from distant sites. Surprisingly, however, no consistent pattern was observed in bacterial phenotypes, genotypes, or protein expression between the upper and lower lobes and no association with disease.

While the studies described above are among several important studies investigating similar issues (11–14), some of which did indeed observe spatial divergence of bacterial communities, these two stood out to me, as each sought to answer tough, passionately debated questions in the field with a direct, thorough, outcome-agnostic approach in patients. As a result, both found surprising results that greatly challenged how I think about chronic polymicrobial infections. The presence of pathogen A or of virulence factor B did not result in measurable damage compared to areas where they were absent, turning my “Koch’s Postulates” notion of infection on its head. Knowledge of precisely how CF pathogens contribute to disease outcomes and how to effectively eradicate them unfortunately remains elusive; however, those studies highlight a need for new ways of considering the pathology of chronic infections in order to improve interventions.
